# Apple pomace as an alternative substrate for butanol production

**DOI:** 10.1186/s13568-023-01649-1

**Published:** 2023-12-06

**Authors:** Olena Tigunova, Viacheslav Bratishko, Sergiy Shulga

**Affiliations:** 1 Institute of Food Biotechnology and Genomics NAS of Ukraine, Laboratory of Food and Industrial Biotechnology, 2a, Baida Vyshnevetskyi Str, Kyiv, 04123 Ukraine; 2 National University of Life and Environmental Science of Ukraine, 15, Heroes Oborony str, Kyiv, 03041 Ukraine

**Keywords:** Butanol, Strain - producer, Apple pomace, Cultivation, *Clostridium*

## Abstract

Butanol-producing strains *Clostridium* sp. UCM B-7570 and *C. acetobutylicum* UCM B-7407 were used for research from “Collection of strains of microorganisms and plant lines for food and agricultural biotechnology” of the Institute of Food Biotechnology and Genomics of the National Academy of Sciences of Ukraine, glycerol (BASF, Germany) and apple pomace (total moisture 4%) after apple juice production. The aim of this work was to study the possibility of using apple pomace by domestic butanol-producing strains of *Clostridium sp.* UCM B-7570 and *C. acetobutylicum* UCM B-7407 as a substrate. Producers were cultured on medium with different concentrations of apple pomace, glycerol was used for the inoculation. The presence of ethanol, acetone, and butanol in the culture liquid was determined using a gas chromatograph. It was determined that a significant part of the macrocomponent composition of the extracts can be used in bioconversion by producing strains of the genus *Clostridium*. It was determined that the highest concentration of butanol (10 g/dm^3^) was at a concentration of 120 g/dm^3^ in the extracts. The obtained data showed the possibility of using apple pomace as a substrate in biobutanol technology.

## Key points

• The highest butanol concentration (10 g/dm^3^) was accumulated using apple pomace.

## Introduction

Ukraine is one of the leaders in the industrial production of apples on the European market (Viotsekhivskyi et al. 2022). The annual volume of apple production in Ukraine is about 700,000 tons, including 250,000 tons of varietal apples for fresh consumption (Salo 2020). About 75% of the apple harvest is processed annually in Ukraine to obtain juice, cider or puree (Sachko et al. 2020). In the process of processing, a large amount of waste is formed - pulp, skin and seeds, which are called pomace (Martau et al. 2021). It is known [5] that during the production of apple juice waste in the form of pomace is 20–30%, puree − 10–18%, compotes − 30–40% (Golebiewska et al. 2022). Industrial waste (pomace) contains valuable compounds such as soluble sugars, structural carbohydrates (cellulose and hemicellulose), minerals and vitamins. Apple pomace (AP), as animal feed, has low nutritional value due to low protein content, so a significant amount of pomace ends up in landfills (Fidriyanto et al. 2023). There is a technology (Putra et al. 2023) for processing pomace to obtain pectin, polyphenols, enzymes, aromatic compounds, antioxidants, organic acids, biopolymers, and biofuel. Among the above-mentioned products, butanol, as one of the types of biofuel, is particularly promising. Butanol has high energy efficiency, low miscibility with water and hygroscopicity, the possibility of use in internal combustion engines without their modernization and is an alternative to fossil fuels (Bravo-Venegas et al. 2023). The energy capacity of butanol is 30% higher than that of ethanol, in addition, it has a lower saturated vapor pressure, which makes it compatible with gasoline in a wide range of ratios (Robles et al. 2023). Currently, butanol is obtained as a result of industrial oil processing or in the process of microbiological synthesis of solventogenic bacteria of the genus *Clostridium* using sugar and starch-containing substrates (Segovia-Hernandez et al. 2022). Due to the rapid increase in food prices, it is relevant to study the accumulation of butanol in the process of acetone-butanol-ethanol (ABE) fermentation on alternative substrates (production waste), such as wheat straw, rice, barley, corn, rapeseed, wire millet, corn cobs (Guo et al. 2022). AP can also be used as an alternative substrate (Ampese et al. 2023). Pomace contain soluble sugars and structural carbohydrates, which makes them a promising substrate for further research on biobutanol technology (Hernandes et al. 2021). The aim of this work was to study the possibility of using AP as a substrate by domestic butanol-producing strains of *Clostridium* sp. UCM B-7570 and *C. acetobutylicum* UCM B-7407.

## Materials and methods

### Bacterial strain and growth media

Butanol-producing strains UCM B-7570 *Clostridium* sp. and *C. acetobutylicum* UCM B-7407 were used for research from “Collection of strains of microorganisms and plant lines for food and agricultural biotechnology” of the Institute of Food Biotechnology and Genomics of the National Academy of Sciences of Ukraine, glycerol (BASF, Germany) and apple pomace (total moisture 4% was obtained after drying in a stationary fruit dryer) after apple juice production. AP had a paste-like consistency and were obtained from Golden Delicious apples (popular industrial variety of apples in Ukraine and Eastern Europe) using a juicer (Philips, the Netherlands). To obtain juice, ripe apple fruits weighing 100–120 g were selected, without damage by pests or diseases, existing external defects, growths, deformations, yellow in color with a skin of medium thickness, dense, elastic, dry with yellowish pulp, dense, fine-grained, juicy, aromatic, sweet-sour taste. The macrocomponent composition of the AP after obtaining the juice from the fruits was determined according to the method (Tigunova et al. 2020). Pectin was removed by filtration after adding water and sterilization of AP.

As an inoculation medium, the following composition (g/l) was used: glycerol – 20; yeast extract – 1; (NH_4_)_2_SO_4_ – 0.6; (NH_4_)_2_HPO_4_ – 1.6; pH 6.5. The medium was sterilized by autoclaving at 1 atm. 30 min. The initial concentration of the applied inoculum was 5% by volume. Different amounts of AP were used as an enzymatic medium for cultivation, KH_2_PO_4_ – 1.5 г/дм^3^, MgSO_4_ × 7H_2_O – 0.2 г/дм^3^, FeSO_4_ × 7H_2_O – 0.01 г/дм^3^, (NH_4_)_2_SO_4_ – the concentration depended on the need, to maintain the C:N balance in the medium and tap water. The medium was sterilized by autoclaving at 0.5 atm. within 30 min.

### Cultivation and solvent determination

Cultivation of microorganisms was carried out using a standard differential enhanced clostridial medium (Condalab, Spain) according to the method (Tigunova et al. 2023) in a “Crystal” anaerostat (Germany) in a nitrogen atmosphere. Cultivation was carried out in 500 ml flasks filled with 250 ml medium and hydrochloric acid modules. The flasks were weighed and thermostated at a temperature of 35 ± 1^0^С. After 72 h of cultivation, the cells were sedimented for 10 min using an ultracentrifuge “Labofuge 400R” (Germany) at a speed of 13,000 rpm, and fermentation products were collected from the culture liquid. Biomass was determined by the weight method (Tigunova et al. 2023). The Presence of ethanol, acetone, and butanol in the culture liquid was determined using a gas chromatograph (a flame ionization detector and a packed column 3 m long, phase - Сarbovax 1500 on the chromaton N-A-W-DMSC (0.20–0.25 mm). The temperature of the column was 60 ± 2^0^ C, the evaporator is160 ± 5^0^ C, the ratio of nitrogen-hydrogen-air flows is 1:1:10. The moisture content of raw materials was determined using a RADWAG MA 50/C/1 weight moisture analyzer (Poland).

### Statistical analysis

Statistical data processing was carried out using the Microsoft Excel 12.0 program. All experiments were performed in triplicate. The difference between the two means was considered significant *р*<0,05.

## Results

### AP macrocomponent composition

**Fig. 1:**
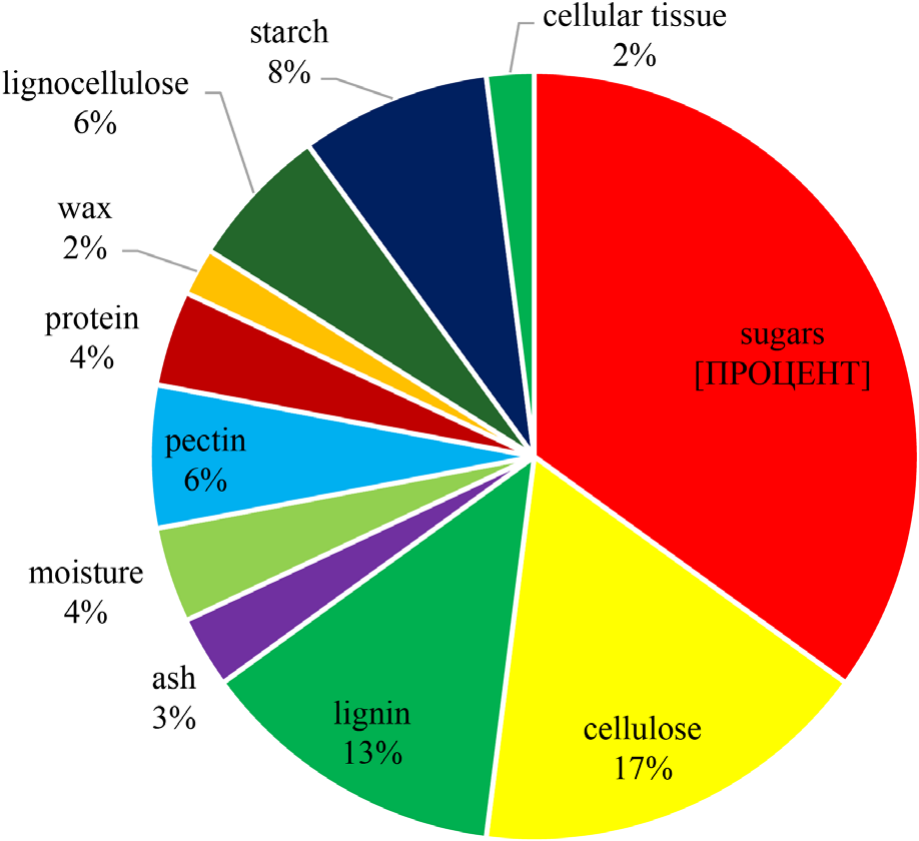
Macrocomponent composition AP of Golden Delicious. AP had a paste-like consistency and were obtained from Golden Delicious apples using a juicer. The macrocomponent composition of the AP after obtaining the juice from the fruits was determined according to the method (Tigunova et al. 2020)

AP mainly consists of peel (50%) and pulp (45%), seeds (2–4%) and stem (1%) (Gumul et al. 2023). The composition of the pomace varies depending on the variety of apples and climatic conditions during cultivation, and therefore the accumulation of butanol in the fermentation process changes (Calvete-Torre et al. 2022). We determined the macro-component composition of the AP of Golden Delicious apples after squeezing the juice. The results of the study are presented in Fig. 1.

It was shown that the largest part of AP consisted of sugars − 35%, cellulose − 17% and lignin − 13%. The rest of the components are represented in smaller quantities - starch 8%, lignocellulose 6%, pectin 6%, protein 4%, moisture 4%, ash 3%, wax 2%.

### Producer cultivation

For cultivation of producer strains of *Clostridium* sp. UCM B-7570 and *C.acetobutylicum* UCM B-7407 AP were used as a complex substrate without prior hydrolysis (Table 1). After fermentation, the strain *Clostridium* sp. UCM B-7570 accumulated 8 g/dm^3^ of butanol and 1.3 g/dm^3^ of ethanol in the culture liquid, while *C. acetobutylicum* UCM B-7407 accumulated 6 g/dm^3^ and 0.9 g/dm^3^, respectively. Acetone and propanediol were present in trace amounts for both strains. The amount of released gases (mixtures of CO_2_ and H_2_) was almost the same in both fermentations (3.8 and 3.5 g/dm^3^, respectively). The conversion of sugars was 85 and 80%, respectively, and the dry residue was 34.15 and 38.6, respectively. The strain *Clostridium* sp. UCM B-7570, which accumulated the highest amount of target product and had the highest sugar conversion with the lowest dry residue was used in further experiments.

**Table 1:** Technological indicators of fermentation of pomace (per 100 g)

Strain	Butanol, g/dm^3^	Ethanol, g/dm^3^	Releaser gases, g/dm^3^	Sugar conversion, %	Dry residue, g
UCM B-7570	8.00 ± 0.01	1.30 ± 0.01	3.80 ± 0.02	85.00 ± 0.05	34.15 ± 0.03
UCM B-7407	6.00 ± 0.01	0.90 ± 0.01	3.50 ± 0.02	80.00 ± 0.05	38.60 ± 0.03

### Inoculation effect

**Fig. 2:**
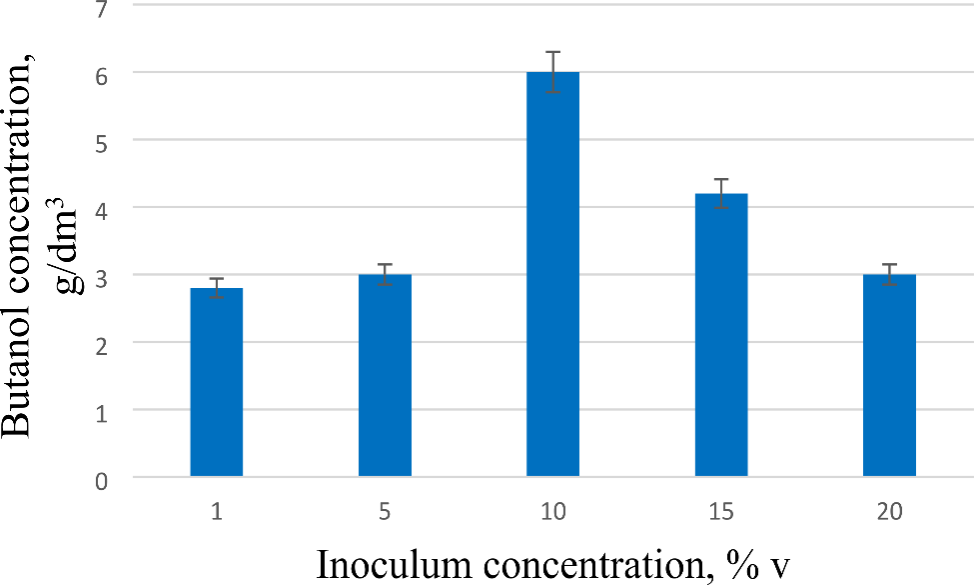
Effect of inoculum on butanol accumulation. As an inoculation medium was used glycerol medium. The concentration of the inoculum had a significate effect on the accumulation of butanol in the enzymatic medium

The amount of inoculum was an important parameter affecting the accumulation of butanol in the culture liquid. Glycerol was used to evenly distribute the concentration of bacteria in the inoculation medium. A study of the effect of the introduced inoculum from the glycerol medium on the accumulation of butanol by the producer-strain UCM B-7570 in the enzymatic medium was carried out (Fig. 2).

As a result of the research, it was shown that the concentration of the inoculum introduced into the seed medium had a significate effect on the accumulation of butanol in the enzymatic medium. With an increase in the inoculum concentration to 15–20% and more, the accumulation of butanol decreased. The optimal concentration of the seed material that was added to the fermentation mixture was determined to be 10%, in this case the highest concentration of butanol was accumulated − 8 g/dm^3^. Further studies were carried out precisely at this concentration of the inoculum.

**Fig. 3:**
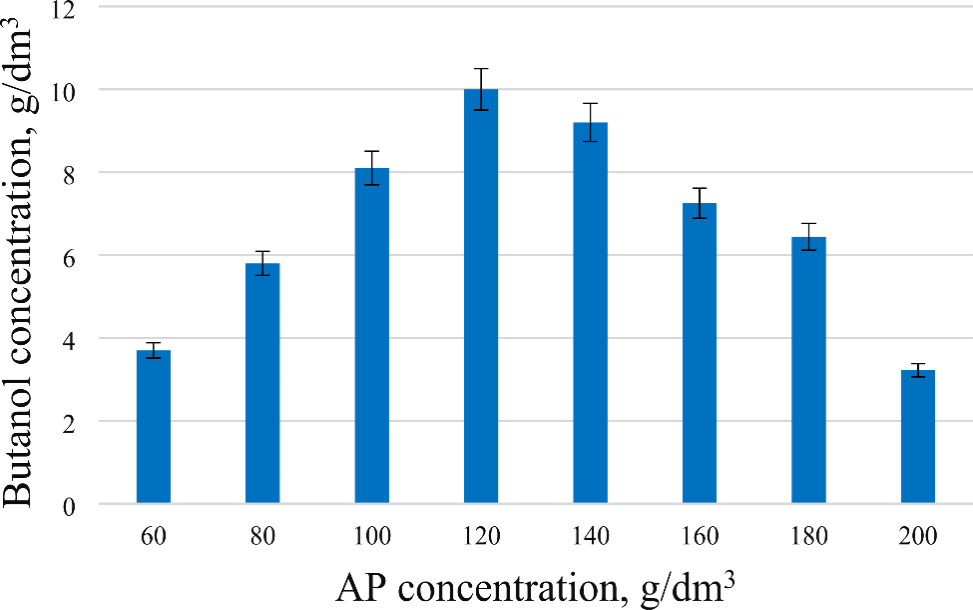
The effect of the AP concentration on the accumulation of butanol. Different amounts of AP were used as an enzymatic medium for cultivation. The accumulation of butanol in the fermentation mixture increased in direct proportion to the increase of the AP concentration to 120 g/dm^3^, but with a further increase the accumulation of butanol decreased

In addition to the effect of the concentration of seed material in the accumulation of butanol, the concentration of the available carbon source also plays a key role. The effect of different biomass concentrations of AP in an enzymatic environment on the accumulation of butanol was studied (Fig. 3).

The obtained data, it can be seen that the accumulation of butanol in the fermentation mixture increased in direct proportion to the increase of the AP concentration from 60 to 120 g/dm^3^, but with a further increase in the concentration from 140 to 200 g/dm^3^, the accumulation of butanol decreased. The highest accumulation of butanol (10 g/dm^3^) was obtained at a concentration of AP of 120 g/dm^3^ in the enzymatic mixture. From the obtained data, it can be assumed that with an increase in the concentration of the carbon substrate, its bioavailability decreases.

## Discussion

Effective waste management is one of the world’s biggest environmental problems. Moreover, there are no signs of this trend slowing down in the near future. The main method of disposal of AP is their transportation to the landfill directly into the soil, but there is also an alternative - the use of pomace in feed and the use of macrocomponents of pomace in technological processes. AP can be used as a complex substrate for microorganisms, which contains many nutrients necessary for their vital activity. The work (Lyu et al. 2020) presents the results of the study of industrial waste in the process of obtaining juice from different varieties of apples. It was shown that the pomace contained 9.0% moisture, 2.27% fat, 2.37% protein, 1.6% ash, 84.7% carbohydrates, 5.6% starch, and 54.2% total sugar, as well as calcium, potassium, and magnesium.

The paper (Jin et al. 2019) shows the possibility of complex use of all (both soluble and insoluble) carbohydrates in AP to obtain butanol. Soluble sugars of AP were extracted with hot water. The lignocellulose-rich residue was pretreated with a dilute acid or alkali solution followed by enzymatic hydrolysis to obtain hydrolyzed sugars (Table 2). Soluble sugars and acid- or alkali-hydrolyzed sugars were combined as a substrate for butanol production by the producer strain *C. beijerinckii* P260 (Jin et al. 2019).

**Table 2:** Accumulation of butanol after hydrolysis

Strains	Substrate treatments method	Butanol concentration, g/dm^3^	Link
*C.beijerinckii* P 260	alkaline and enzymatic hydrolysis	7.03	Jin et al. (2019)
	acid and enzymatic hydrolysis	7.16	Jin et al. (2019)
* C.beijerinckii* СЕСТ 508	with surfactants and enzymatic hydrolysis	9.11	Hijosa-Valsero et al. (2017)
	enzymatic hydrolysis	10.75	Bravo-Venegas et al. (2023)
* C.beijerinckii* NRRL B-466	acid hydrolysis and ultrafiltration	9.3	Maiti et al. (2016)
* C. beijerinckii* ВА 101	mixtures with other agro-industrial waste	9.2	Jesse et al. (2002)
* C.beijerinckii* NRRL B-592	hydration	8.52	Voget et al. (1985)
* C.beijerinckii* NRRL B-593	hydration	8.32	Voget et al. (1985)
* C.acetobutylicum* NRRL B-596	hydration	7.41	Voget et al. (1985)
* C.acetobutylicum* UCM B-7407	hydration	6.0	This work
*Clostridium* sp. UCM В-7570	hydration	10.0	This work

Other authors (Voget et al. 1985) used only soluble sugars of pomace for butanol production. In the work (Hijosa-Valsero et al. 2017), the authors applied various types of pretreatment (autohydrolysis, acid hydrolysis, alkaline hydrolysis, hydrolysis with organic solvents and surfactants) of pomace with subsequent enzymatic hydrolysis to split structural carbohydrates into hydrolyzed sugars and obtain butanol.

The macrocomponent composition of the apple juice of the Golden Delicious variety determined by us (Fig. 1) differed from the macrocomponent composition of the Papirovka variety apples, which is given in the previous work of the authors (Tigunova and Shulga 2021), where the percentage of sugar was 39.2; cellulose – 16.6; lignin – 12.8; ash − 2.6; moisture – 4.2; pectin − 11.9; protein − 4.5; wax − 1.7; lignocellulose − 6.5.

The obtained results make it possible to conclude that most of the AP components can be used by producer strains of the genus *Clostridium* in bioconversion without prior hydrolysis. The exceptions were lignin and wax, which were not fermented, and pectin, fiber, and hemicelluloses, which were only partially fermented. After extraction, pectin can be used in food industry products (Borujeni et al. 2023; Luo et al. 2023; Naqash et al. 2021; Giron-Hernandez et al. 2023).

The possibility of conversion by strains UCM B-7570 and UCM B-7407 is shown as a result of the study of an alternative substrate (AP) without hydrolysis of the macrocomponents of pomace with the production of butanol. The macrocomponent composition of the substrate showed that most of it can be used by microorganisms to accumulate the target product - butanol. It was shown that the producer strain UCM B-7570 converted a higher percentage of sugars of AP during cultivation. It was determined that the highest concentration of butanol (10 g/dm^3^) was accumulated when adding 10% inoculum and at a concentration of 120 g/dm^3^ of the substrate. The obtained data show the possibility of using AP as an alternative substrate in biobutanol technology.

## Data Availability

All data generated or analyzed during this study are included in this published article.

## References

[CR1] Ampese LC, Ziero HDD, Velasquez J, Sganzerla WG, Martins G, Forster-Carneiro T (2023). Apple Pomace management by anaerobic digestion and composting: a life cycle assessment. Biofpr.

[CR2] Borujeni NE, Alavijeh MK, Denayer JFM, Karimi K (2023). A novel integrated biorefinery approach for apple pomace valorization with signification socioeconomic benefits. Renew Energy.

[CR3] Bravo-Venegas J, Prado-Acebo I, Gullon B, Lu-Chau TA, Eibes G (2023). Avoiding acid Crash: from apple pomace hydrolysate to butanol through acetone-butanol-ethanol fermentation in a zero-waste approach. J Waste Manag.

[CR4] Calvete-Torre I, Sabater C, Anton JM, Moreno FJ, Riestra S, Margolles A, Ruiz L (2022). Prebiotic potential of apple pomace and pectins from different apple varieties: modulatory effects on key target commensal microbial populations. Food Hydrocoll.

[CR5] Fidriyanto R, Singh PB, Manju KM, Widyastuti Y, Goel G (2023) Multivariate analysis of structural and functional properties of fibers from apple pomace using different extraction methods. Food Prod Process and Nutr 5(6). 10.1186/s43014-022-00119-8

[CR6] Giron-Hernandez J, Pazmino M, Barrios-Rodriguez YF, Turo CT, Wills C, Cucinotta F, Benlloch-Tinico M, Gentile P (2023). Exploring the effect of utilising organic acid solutions in ultrasound-assisted extraction of pectin from apple pomace, and its potential for biomedical purposes. Heliyon.

[CR7] Golebiewska E, Kalinowska M, Yildiz G (2022). Sustainable use of apple pomace (AP) in different industrial sector. J Mater.

[CR8] Gumul D, Kruczek M, Ivanisova E, Slupski J, Kowalski S (2023). Apple Pomace as an ingredient enriching wheat pasta with health-promoting compounds. Foods.

[CR9] Guo Y, Liu Y, Guan M, Tang H, Wang Z, Lin L, Pang H (2022). Production of butanol from lignocellulosic biomass: recent advances, challenges, and prospects. RSC Adv.

[CR10] Hernandes D, Rebolledo-Leiva R, Fernandez-Puratich H, Quinteros-Lama H, Cataldo F, Munoz E, Tenreiro C (2021). Recovering apple agro-industrial waste for bioethanol and vinasse joint production: screening the potential of Chile. Fermentation.

[CR11] Hijosa-Valsero M, Paniagua-Garcia IA, Diez-Antolinez R (2017). Biobutanol production from apple pomace: the importance of pretreatment methods on the fermentability of the lignocellulosic agro-food waste. Appl Microbiol Biotechnol.

[CR12] Jesse TW, Ezeji TC, Qureshi N, Blaschek HP (2002). Production of butanol from starch-based waste packing peanuts and agricultural waste. J Ind Microbiol Biotechnol.

[CR13] Jin Q, Quereshi N, Wang H, Hung H (2019). Acetone-butanol-ethanol (ABE) fermentation of soluble and hydrolyzed sugars in apple pomace by *Clostridium beijerinckii* P260. Fuel.

[CR14] Luo S, Wang S, Yang X, Yuan K, Zhang H, Zhang S, Yang X, Guo Y (2023). Gelation behaviors and mechanism of a new pectic polysaccharide from apple pomace as a potential gelatin substitute. Int J Biol Macromol.

[CR15] Lyu F, Luiz FS, Azeredo PRD, Cruz GA, Ajlouni S, Ranadheera SC (2020). Apple Pomace as a functional and healthy ingredient in food products: a review. Processes.

[CR16] Maiti S, Sarma SJ, Brar SK, Bihan YL, Drogui P, Buelna G, Verma M (2016). Agro-industrial wastes as feedstock for sustainable bio-production of butanol by *Clostridium beijerinckii*. FBP.

[CR17] Martau GA, Teleky B-E, Ranga F, Pop ID, Vodnar DS (2021) Apple Pomace as a sustainable substrate in sourdough fermentation. Sec Food Microbiol 12. 10.3389/fmicb.2021.742020 10.3389/fmicb.2021.742020 PMC8714949 34975780

[CR19] Naqash F, Masoodi FA, Gani A, Nazir S, Jhan F (2021). Pectin recovery from apple pomace: physico-chemical and functional variation based on methyl-esterification. Food Sci + Technol.

[CR18] Putra NR, Rizkiyah ND, Aziz AHA, Yunus CAM, Veza I, Harny I, Tirta A (2023). Waste to wealth of apple pomace valorization by past and current extraction processes: a review. Sustainability.

[CR20] Robles A, Sundar VS, Rangan SM, Delgado AG (2023) Butanol as a major product during ethanol and acetate chain elongation. Front Bioeng Biotechnol 11. 10.3389/fbioe.2023.1181983 10.3389/fbioe.2023.1181983 PMC10233103 37274171

[CR21] Sachko A, Kobasa I, Moysyura O, Vorobets M (2020). Efficiency of apple juice clarification with using of nano-sized mineral oxides. Ukr Food J.

[CR22] Salo IA (2020). Current situation and forecast of the apple market conjuncture in Ukraine. Ekon APK.

[CR23] Segovia-Hernandez J, Behera S, Sanchez-Ramirez E (2022) Advances and development in biobutanol production. Imprint: Woodhead Publishing. 404 p. https://shop.elsevier.com/books/advances-and-developments-in-biobutanol-production/hitchen/978-0-32391178-8

[CR26] Tigunova OO, Shulga SM (2021) Butanol accumulation by butanol strains producers using apple pomace. Proceedings XVI International SummerSchool Conference: Biology, Biotechnology, Biomedicine. Odesa. 63 https://www.researchgate.net/publication/356252504_Butanol_accumulation_by_butanol_strains_producers_using_apple_pomace

[CR25] Tigunova OO, Kamenskyh DS, Tkachenko TV, Yevdokymenko VA, Kashkovskiy VI, Rakhmetov DB, Blume YB, Shulga SM (2020). Biobutanol production from plant biomass. Open Agric J.

[CR24] Tigunova OO, Bratishko VV, Shulga SM (2023). An increase in the production of butanol by *Clostridium* sp. cell under the influence of stress factors. Cyt Gen.

[CR27] Viotsekhivskyi V, Poshkebnov V, Shish A, Matviienko A, Smetanska I, Kusnetsov A, Maliarchuk O, Svinarchuk O, Yushkevich M (2022) Rating and economic assessment of late apples. SWorldJournal. 12 (10): 11–15 10.30888/2663-5712.2022-12-01-006

[CR28] Voget CE, Mignone CF, Ertola RJ (1985). Butanol production from apple pomace. Biotechnol Lett.

